# Clinical, Morphological, and Molecular Study on Grade 2 and 3 Pleomorphic Xanthoastrocytoma

**DOI:** 10.3390/curroncol30020183

**Published:** 2023-02-16

**Authors:** Hui Zhang, Xiao-Jing Ma, Xue-Ping Xiang, Qi-Yuan Wang, Jin-Long Tang, Xiao-Yan Yu, Jing-Hong Xu

**Affiliations:** 1 Departments of Clinical Pathology, The Second Affiliated Hospital of Medical College of Zhejiang University, 88 Jiefang Road, Hangzhou 310003, China; 2 Departments of Clinical Radiography, The Second Affiliated Hospital of Medical College of Zhejiang University, Hangzhou 310003, China

**Keywords:** pleomorphic xanthoastrocytoma, *TERT* promoter mutations, *BRAF V600E* mutation, malignant progression

## Abstract

Purpose: Pleomorphic xanthoastrocytoma (PXA) is an uncommon astrocytoma that tends to occur in children and young adults and has a relatively favorable prognosis. The 2021 WHO classification of tumors of the central nervous system (CNS WHO), 5th edition, rates PXAs as grade 2 and grade 3. The histological grading was based on mitotic activity (≥2.5 mitoses/mm^2^). This study specifically evaluates the clinical, morphological, and, especially, the molecular characteristics of grade 2 and 3 PXAs. Methods: Between 2003 and 2021, we characterized 53 tumors with histologically defined grade 2 PXA (n = 36, 68%) and grade 3 PXA (n = 17, 32%). Results: Compared with grade 2 PXA, grade 3 PXA has a deeper location and no superiority in the temporal lobe and is more likely to be accompanied by peritumoral edema. In histomorphology, epithelioid cells and necrosis were more likely to occur in grade 3 PXA. Molecular analysis found that the *TERT* promoter mutation was more prevalent in grade 3 PXA than in grade 2 PXA (35% vs. 3%; *p* = 0.0005) and all mutation sites were C228T. The cases without *BRAF V600E* mutation or with necrosis in grade 3 PXA had a poor prognosis (*p* = 0.01). Conclusion: These data define PXA as a heterogeneous astrocytoma. Grade 2 and grade 3 PXAs have different clinical and histological characteristics as well as distinct molecular profiles. *TERT* promoter mutations may be a significant genetic event associated with anaplastic progression. Necrosis and *BRAF V600E* mutation play an important role in the prognosis of grade 3 PXA.

## 1. Introduction

Categorized in circumscribed astrocytic gliomas in the 2021 WHO classification of tumors of the central nervous system, 5th edition (CNS WHO), pleomorphic xanthoastrocytoma (PXA) predominantly affects children and young adults [1,2]. PXA is usually located in the cerebral hemisphere, mostly in the temporal lobe, and tends to grossly appear as a superficial cystic and solid mass [3]. Grade 2 PXA has a relatively favorable prognosis [1,3,4]. Grade 3 PXA is diagnosed solely on the basis of tumor histopathologic features and requires increased proliferative activity, the count of ≥5 mitoses per 10 high-power fields (corresponding to 4 mm^2^). Grade 3 PXA manifests both at initial diagnosis and at recurrence. In one series, anaplasia was present in 31% of the cases at first diagnosis [5]. The anaplastic progression of PXA may demonstrate less pleomorphism, more diffuse infiltration, and more necrosis, with higher mitotic activity, than typical classic grade 2 PXA in histological features and leads to a significantly worse survival [1,6,7]. 

PXAs are characterized by a high frequency of mitogen-activated protein kinase (MAPK) pathway alterations. The *BRAF* point mutation rates in PXA reported in the literature range from 60% to 78% [4,8], and most are the V600E type. Other MAPK alterations have been identified in a smaller fraction of PXA, including *BRAF* insertion/deletion mutations [9]. Homozygous 9p21.3 deletions involving the *CDKN2A/B* loci have been identified in grade 2 and 3 PXAs. Initial studies identified this gene homozygous deletion in approximately 60% of the cases [10], while more recent reports have found a higher incidence (>85%) [8,9,11].

In grade 3 PXA, recent studies detected telomerase reverse transcriptase (*TERT*) alteration promoter mutation in 7 of 15 cases (47%), with gene amplification (n = 2) or promoter hotspot mutation (n = 5). However, the cohort of grade 2 tumors was too small (n = 4) to compare grade 2 and grade 3 tumors [11]. *TERT* promoter mutations were identified in 2.0% (1/53) of grade 2 and 14.3% (2/14) of grade 3 PXAs [9]. Although we have more clarity regarding certain molecular events in PXA, whether there are molecular markers associated with anaplastic progression is yet to be confirmed. To better understand the biological characteristics of grade 2 and grade 3 PXAs, we detected and compared the expression of conventional genes and clinical and morphological features in 37 grade 2 PXAs and 20 grade 3 PXAs including the recurrence samples. Our results confirm that the majority of PXAs are characterized by MAPK activation. Necrosis and *BRAF V600E* mutation may be important prognostic factors.

## 2. Methods

### 2.1. Case Selection

In this retrospective study, a total of 57 tumors (37 grade 2 PXAs and 20 grade 3 PXAs) from 53 patients were received from the Second Affiliated Hospital of Zhejiang University, Hangzhou, China, from 2003 to 2021. In three of the patients, tumors had recurred. Two senior neuropathologists reviewed and confirmed all tissue samples from primary or recurrent tumors to represent the PXAs according to the World Health Organization (WHO) classification of tumors of the central nervous system. All samples were routinely formalin-fixed and paraffin-embedded. A final diagnosis was made on the basis of histopathological examinations, immunohistochemical (IHC) analyses, and molecular tests.

### 2.2. DNA Extraction

Each tumor sample used for DNA extraction was histologically verified to contain vital PXA tissue with an estimated tumor cell content ≥80%. Samples were obtained as formalin-fixed, paraffin-embedded (FFPE) tissue (five sections, each 7 µm thick) from patients with PXA. Genomic DNA was extracted using the DNA FFPE Tissue Kit (Amoy Diagnostics Co., Ltd., Xiamen, China). DNA purity and quantification were assessed using the NanoDrop 2000 UV–Vis spectrophotometer (NanoDrop products, Wilmington, DE, USA).

### 2.3. BRAF V600E Analysis

*BRAF* V600E analysis was performed on an MX3000p real-time PCR system with *BRAF* V600E Diagnostic Kit (Amoy Diagnostics Co., Ltd.), according to the manufacturer’s protocol.

### 2.4. TERT Promoter Mutation Analysis

Sanger sequencing was used to analyze the mutation hotspots in the *TERT* promoter. Mutations in the *TERT* promoter had previously been identified mainly as C228T and C250T. For the analysis, the *TERT* promoter mutation analysis kit (Sinomdgene Co., Ltd., Beijing, China) was used according to the manufacturer’s protocol.

### 2.5. Fluorescence In Situ Hybridization

*CDKN2A*, *EGFR*, and *PTEN* statuses were identified via fluorescence in situ hybridization (FISH), using the *CDKN2A* (9p21), the *EGFR* (7p11), and *PTEN* (10q23) probes (ABP Medicine Science and Technology Co., Ltd., Guangzhou, China). An abnormal probe signal counted as at least 15% of the nucleus, and an abnormal signal counted a minimum of 20 consecutive non-overlapping tumor nuclei.

## 3. Statistical Analysis 

Patient characteristics between groups were compared using the chi-square test as appropriate. Cumulative survival probabilities were estimated using the Kaplan–Meier method. The log rank test was used to compare survival across groups. Overall survival (OS) was defined as the duration from the date of initial surgery to that of either death or the last follow-up, with a censoring cutoff date of 30 November 2021 without accounting for eight patients who were lost to follow-up (cases 29 to 34 and cases 52 and 53). Patients alive at the last follow-up were considered censored during the survival analysis. In all analyses, *p*-values < 0.05 were considered statistically significant. 

## 4. Results

### 4.1. Patient Demographics

Table 1 summarizes the clinical features of PXA. It occurs equally in male and female patients and typically develops in children and young adults. Children have no obvious superiority in incidence rate. The temporal lobe was the most common site of PXA. Grade 2 PXA more often involves the cerebral cortex; epilepsy symptoms are more common in patients. In all, eight patients were lost to follow-up, and our data do not contain the surgical status of the 8 patients at the time because some patients were considerably aged. In addition, 13 patients did not have enhanced cranial MRI at our medical center. 

In our series, most of our grade 3 PXA cases were de novo. Only in three cases did the grade 3 tumor recur: in cases 19 and 22, the tumors recurred with anaplastic transformation after incomplete resection and following radiation therapy in 6 months and 18 months, respectively, and in case 33, the tumor recurred 30 months later, without any treatment, after total tumor resection.

### 4.2. Morphologic Patterns of PXA

**Figure 1:**
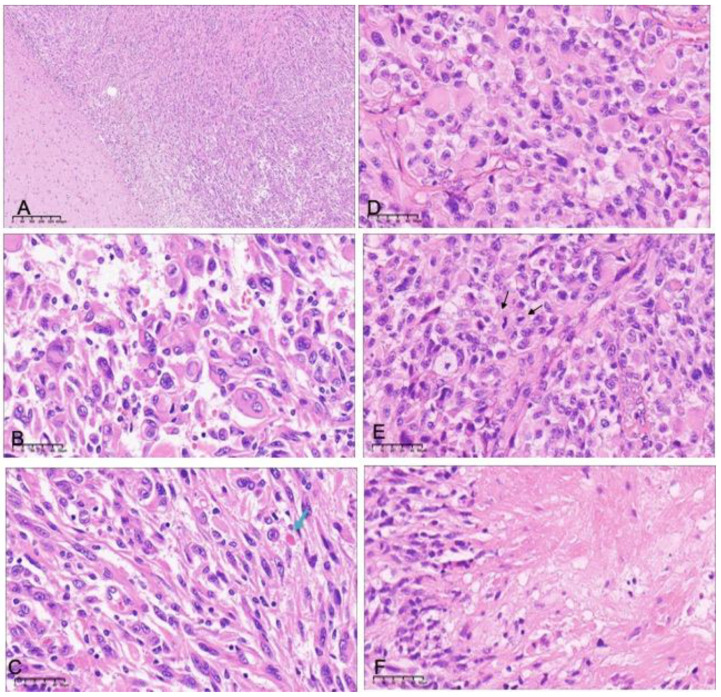
Histopathologic features of grade 2 (**A**–**C**) and grade 3 (**D**–**F**) pleomorphic xanthoastrocytoma. (**A**) There is a clear boundary between the tumor and the brain tissue in grade 2 PXA (H&E ×40). There is an admixture of spindle cells and neoplastic cells with bizarre nuclei or multinucleation (**B**) and eosinophilic granular bodies (**C**, arrows) (H&E ×400). Grade 3 pleomorphic xanthoastrocytoma showing (**D**) a more pronounced epithelioid component, (**E**) high levels of mitotic activity (arrows), and (**F**) focal necrosis (H&E ×400).

The histological findings are summarized in Table 2. In this study, most of the tumors had boundaries, and a few cases presented infiltrating growth patterns (Figure 1A). The neoplastic cells were pleomorphic and multinucleated (Figure 1B). Epithelial cells were more present in grade 3 PXA (*p* = 0.0003). Lymphoid cuffs and xanthomatous change could be seen in almost all cases. Some cases showed eosinophilic granular bodies (EGB) (Figure 1C). We found non-palisading (focal) necrosis in only four grade 3 PXA cases (Figure 1F). Microvascular proliferation and palisading necrosis were not seen in any of the cases. In some of the primary cases of grade 3 PXA, the typical morphology of grade 2 PXA could be seen around the tissue with anaplastic features. However, in grade 3 PXA, eosinophilic bodies (*p* = 0.01) and xanthomatous cells (*p* = 0.04) were reduced to varying degrees compared with grade 2 PXA. 

### 4.3. Molecular Results

**Figure 2:**
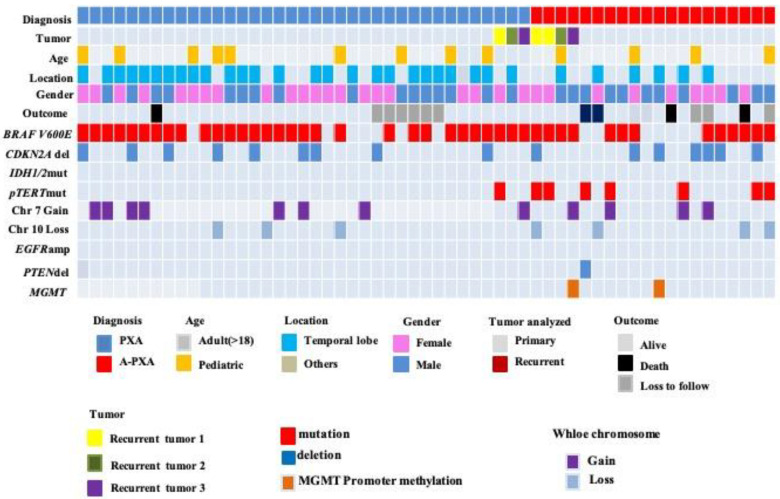
Clinical and molecular characteristics of the patient cohort. *BRAF V600E* mutations were identified by immunohistochemistry (IHC) or real-time polymerase chain reaction (PCR). *MGMT* promoter methylation status was assessed by methylation-specific real-time PCR system with an *MGMT* methylation analysis kit. *TERT* promoter mutations were identified by Sanger sequencing, and all of the mutation sites were C228T. *CDKN2A/B*, *EGFR*, and *PTEN* statuses were identified via fluorescence in situ hybridization (FISH).

Table 3 and Figure 2 summarize the molecular detection results. *BRAF* V600E mutation was the most frequent gene alteration in our series, not related to PXA histological grade (*p* = 0.18). The mutant status of *BRAF* V600E in our three relapse cases did not change with or without treatment. Most BRAF mutation-positive cases occurred in the temporal lobe (25/43, 58%). 

**Figure 3:**
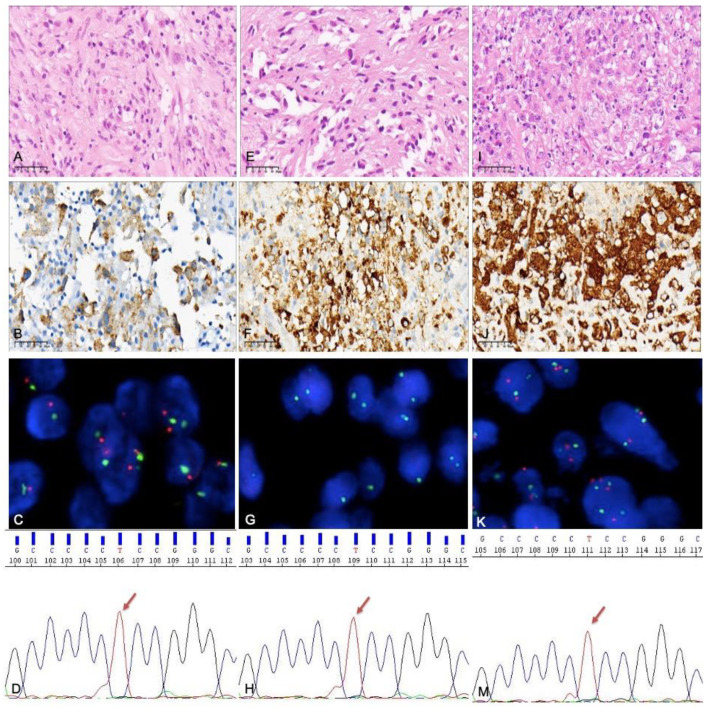
Histological molecular examination results for a tumor that recurred twice after incomplete excision: (**A**–**D**) case 19 (the primary tumor), (**E**–**G**) case 46 (the first relapse), and (**I**–**K**,**M**) case 53 (the second relapse). (**A**) An area composed of mostly spindle cells and some multinucleated cells. (**B**) *BRAF V600E* expressed by tumor cells (by immunohistochemistry). (**C**) *CDKN2A* identified via FISH, with no homozygous deletion. (**D**) *TERT* promoter mutation detected by PCR; the site was C228T. (**E**) Spindle cell areas can still be seen in the tumor with first recurrence. (**F**) *BRAF V600E* expressed by tumor cells. (**G**) *CDKN2A* homozygous deletion identified via FISH. (**H**) *TERT* promoter mutation; the site was C228T. (**I**) Epithelioid cells as the main component, accompanied by significant nuclear atypia. (**J**) *BRAF V600E* expressed by tumor cells. (**K**) There was no *CDKN2A* homozygous deletion. (**M**) *TERT* promoter mutation; the site was still C228T.

*TERT* promoter mutation has obvious advantages in grade 3 PXA; only one was detected in grade 2 PXA. All of the mutation sites were C228T. Cases 19, 46, and 53 were diagnosed as grade 2, grade 3 (the first recurrence), and grade 3 (the second recurrence) PXA, respectively (all three tumors from the same patient). The tumor was in the corpus callosum and underwent an incomplete resection. In the three cases, both *TERT* promoter C228T and *BRAF* V600E mutations were constantly present, while *CDKN2A* homozygous deletion was only seen in the first recurrence (case 46) (Figure 3). 

The proportion of *CDKN2A* homozygous deletion in our cases was 30.2% (n = 16), and there was no significant association between grade 2 and grade 3 PXA. None of the cases had *IDH1/2* gene mutations. Methylation of the *MGMT* promoter occurred only in two cases of grade 3 PXA. The chromosome 7 gain was also detected in grade 2 PXA (n = 7) and grade 3 PXA (n = 5). The chromosome 10 loss was discovered in grade 2 PXA (n = 3) and grade 3 PXA (n = 4). The results were not associated with tumor grade or other molecules. No tumors demonstrated concurrent 7 gain/10 loss, characteristically found in IDH-wildtype glioblastoma.

In our results, there were more molecular change events in grade 3 than in grade 2 PXA. In our cohort, the simultaneous occurrence of ≥3 molecular changes in one case was considered a multimolecular change event. A comparison between the two groups of data shows that *p* = 0.07.

## 5. Survival

**Figure 4:**
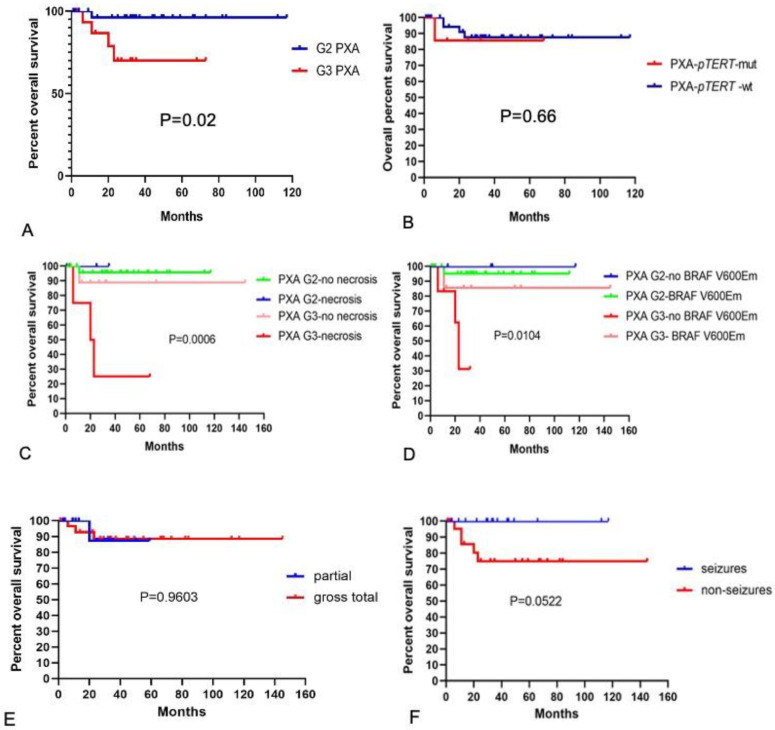
Overall survival of PXA. (**A**) Overall survival for grade 2 and 3 PXA. The blue line shows grade 2 PXA, and the red line depicts grade 3 PXA. The differences between the two groups were statistically significant (*p* = 0.02). (**B**) Overall survival in the case of PXA with or without *pTERT* mutation. The blue line depicts PXA without *pTERT* mutation, and the red line depicts PXA with *pTERT* mutation. The differences between the two groups were not statistically significant (*p* = 0.66). (**C**) Overall survival of different grades of PXA with and without necrosis. (**D**) Overall survival of different grades of PXA with and without BRAF V600E mutation. (**E**) Overall survival of different extension of resection. (**F**) Overall survival of PXA with and without epileptic symptoms. *M*, *mut*, mutation; *wt*, wildtype.

Kaplan–Meier survival analysis of 45 PXA cases highlighted the differences in the survival curves of grade 2 and 3 tumors. The overall survival of grade 3 PXA cases was lower than that of grade 2 PXA cases (*p* = 0.02), demonstrating that the histologic grade was a robust predictor of overall tumor survival (Figure 4A). There was no statistical difference in the overall survival of the cases with *TERT* promoter mutations compared with those without mutations (Figure 4B). Cases with histological features of necrosis also showed poorer overall survival in grade 3 PXA (Figure 4C), and tumors without *BRAF V600E* mutation in grade 3 PXA showed a lower survival rate (Figure 4D). There was no statistical difference in overall survival of cases with different extension of resection (Figure 4E); the survival curves of PXA with seizures were slightly higher than those without seizures in our cohort (Figure 4F).

## 6. Discussion

Grade 3 PXA may occur de novo at first resection or may evolve from PXA grade 2. Reports suggest that grade 3 PXAs are more likely to occur primarily or to recur mostly in adults [10,12]. In our cohort, most grade 3 cases were de novo. The temporal lobe was the most prevalent site of PXA [1,3,5,6,13], and we found that grade 3 PXA bias involved white matter other than cortex in the brain and was more likely to occur beyond the temporal lobe than grade 2 PXA. Grade 3 PXAs were more likely to present imaging features of surrounding edema than grade 2 PXAs in MRI, which is one of the imaging features of high-grade glioma. Epilepsy was more common in grade 2 PXAs due to the superficial location. Studies revealed that epileptic seizures are an independent and positive prognostic factor for low-grade glioma [14,15]. In line with our data, the survival curve of PXA with seizures was slightly higher than that without seizures in our cohort. Extent of resection is the most significant prognostic factor associated with recurrence, but not of overall survival [5,6,16], consistent with our results.

EGBs, perivascular lymphocytes, xanthic cells, and multinucleated cells are histological features of classic PXA [9,13] and mainly occur in grade 2 PXAs. Grade 3 PXAs may demonstrate less pleomorphism and a more diffusely infiltrative pattern than their grade 2 counterparts. Histological grade is an important prognostic indicator recognized by the WHO classification of CNS tumors. Kaplan–Meier survival analysis highlighted the differences in survival curves of grade 3 tumors with and without necrosis. However, necrosis in grade 2 tumors was not associated with different outcome. In this case, the low number of cases with outcome data may compromise this distribution. Some studies suggested that necrosis is also a significant predictor of overall survival, but it is still difficult to detect a difference in survival between patients whose tumors had high mitotic counts and necrosis versus those with only necrosis [5].

*BRAF V600E* mutation can be found in 70% of typical PXAs; the frequency is lower in grade 3 PXA than in grade 2 PXA [1,8,17]. The temporally located PXAs also tend to harbor *BRAF* mutations [6]. The presence of *BRAF V600E* mutation in grade 3 PXAs was associated with better prognosis in our cohort. Studies reported in the literature that patients with *BRAF V600E* mutant tumors had significantly longer overall survival when compared with those without *BRAF V600E* mutant tumors [5,18,19]. *BRAF V600E* mutation and further MAPK activating molecular alterations are also frequent events in low-grade glial and glioneuronal tumors [8,12,20,21]. The prognostic impact of *BRAF* mutation is still a matter of debate and requires further studies on the frequency of all MAPK activating alterations in PXAs.

In many studies, *CDKN2A/B* homozygous deletion has been identified in PXA. However, the frequency at which it has been observed has varied across studies, from approximately 60% up to 100% [8,9,10,11]. This variability in detection sensitivity has important implications, as *CDKN2A/B* is increasingly used as a diagnostic and prognostic marker [9]. In IDH-mutant astrocytomas, *CDKN2A/B* homozygous deletion is an adverse prognostic factor. However, there was no difference in the expression between grade 2 and 3 PXAs, nor was there any prognostic implication. Our data showed *CDKN2A* deletion in grade 2 PXA (26%, n = 9) and grade 3 PXA (37%, n = 7). The *CDKN2A* deletion rate in our series was lower [8,11]. Next-generation sequencing of 295 cancer-related genes was used to investigate the molecular profiles of 13 cases of PXA in China. The results showed *CDKN2A/B* homozygous deletion only in one case [22]. There may be some differences in gene expression in patients with different ethnicities. In addition, FISH was used to detect the CDKN2A gene in our series. The NGS method used in the studies is more sensitive for gene detection, and the long storage time of blocks in some cases may be one of the factors affecting the quality and results of FISH detection. At present, there is no large case-series study in China on the homozygous deletion ratio of *CDKN2A/B* in PXA. More research data are needed for comprehensive and objective analysis.

The *TERT* gene plays an important role in the malignant progression of astrocytic gliomas [20,23,24,25,26,27]. According to the data reported in the literature, altered *TERT* gene status changes were present in approximately 47% of grade 3 PXA cases, including *TERT* promoter hotspot mutation and *TERT* gene amplification [11]. In our cohort, *TERT* promoter mutations were detected in seven grade 3 PXA (7/20, 35%) cases. Only one grade 2 PXA case was identified with a *TERT* promoter mutation, but the tumor recurred as grade 3 PXA within half a year. Our results suggest that compared with grade 2 PXA, grade 3 PXA is more prone to *TERT* gene alteration (*p* = 0.005). Grade 2 PXA with *TERT* gene alteration is more likely to undergo malignant transformation [28]. Our study was limited by the small number of patients with *TERT* promoter mutation and by the imperfect follow-up. It could only be confirmed that *TERT* promoter mutations are more common in grade 3 tumors; no effect of *TERT* promoter mutation on prognosis was observed. A recent article reported the presence of canonical *TERT* promoter mutations as a robust indicator for poor prognosis in methylation class PXA (mcPXA). Their results suggest that histologically, PXA and mcPXA may be different entities [18]. We suggest that alterations in the *TERT* promoter may be one of the molecular diagnostic criteria for grade 3 PXA.

We also found some cases with a chromosome gain +7 (n = 12) and a chromosome loss −10 (n = 7). None of the cases presented *EGFR* gene amplification, and only one case showed *PTEN* gene deletion. These results were not associated with tumor grade or other molecular features, and no tumors demonstrated a concurrent gain +7/loss −10, which is characteristically found in IDH-wild type glioblastoma. This also indicates, to some extent, that the molecular characteristics of grade 3 PXA are different from those of IDH-wildtype glioblastoma.

*MGMT* promoter methylation occurs in 40% of primary glioblastoma cases and is associated with increased survival after radiotherapy and chemotherapy with temozolomide [29]. In our series, *MGMT* promoter hypermethylation was found only in two cases of grade 3 PXAs (3.5%) and emphasized that *MGMT* promoter hypermethylation is a rare epigenetic event in PXA.

In summary, PXA is a heterogeneous entity. Our data demonstrate that *TERT* promoter mutations have the highest occurrence in grade 3 PXA and suggest that these mutations may contribute to anaplastic progression and that necrosis and absence of *BRAF V600E* mutation within grade 3 PXAs are associated with poor prognosis. 

## Figures and Tables

**Table 1:** Clinical pathological features of PXA.

Clinical Pathological Features	PXA G2 n (%)	PXA G3 n (%)	ALL, n (%)	*p* Value
Total numbers of patients	36	17	53	
Gender				0.25
Male	15 (42%)	10 (59%)	25 (47%)	
Female	21 (58%)	7 (41%)	28 (53%)	
Age at diagnosis (years)				
Median	27 (5–59)	37 (13–74)	32 (5–74)	
≤18 y	9 (11%)	4 (5%)	13 (9%)	0.9
>18 y	27 (89%)	13 (95%)	40 (91%)	
Location				
Temporal lobe	26 (72%)	6 (35%)	32 (60%)	0.009 *
Non-temporal lobe	10 (28%)	11 (65%)	21 (40%)	
Radiology				
Contrast enhancement				0.08
Present	15 (63%)	14 (88%)	29 (72%)	
Absent	9 (37%)	2 (12%)	11 (28%)	
Cystic changes				0.36
Present	13 (54%)	11 (69%)	24 (40%)	
Absent	11 (46%)	5 (31%)	16 (40%)	
Surrounding edema				0.01 *
Present	6 (25%)	10 (63%)	16 (40%)	
Absent	18 (75%)	6 (37%)	24 (60%)	
Superficial				0.001 *
Present	24 (100%)	10 (63%)	34 (85%)	
Absent	0	6 (37%)	6 (15%)	
Seizures				
Present	17 (47%)	3 (18%)	20 (37%)	0.04 *
Absent	19 (51)	14 (82%)	33 (63%)	
Extent of surgery				
Gross total resection	20 (71%)	12 (71%)	32 (60%)	0.85
Subtotal resection	8 (29%)	5 (29%)	13 (40%)	

*p*-Values compare grade 2 and grade 3 tumors. Bold values and * are statistically significant variables. Non-temporal lobe in location refers to parietal lobe, occipital lobe, amygdala, corpus callosum, lateral ventricle, basal ganglia in the series. Superficial refers to the tumor cortical involvement.

**Table 2:** Histological features of PXA.

Histological Features	PXA G2 n (%)	PXA G3 n (%)	ALL n (%)	*p*-Value
Total number of tumors	37	20	57	
Predominant cell type				
Spindle cells	31 (84%)	12 (60%)	43 (75%)	0.11
Pleomorphic (multinucleated) cells	6 (16%)	5 (25%)	11 (19%)	0.23
Epithelioid cells				0.0003 *
Present	6 (22%)	12 (75%)	18 (42%)	
Absent	21 (78%)	4 (25%)	25 (58%)	
Multinucleated cells				0.03 *
Present	35 (94%)	15 (75%)	50 (88%)	
Absent	2 (6%)	5 (25%)	7 (12%)	
Xanthoma cells				0.04 *
Present	25 (67%)	7 (35%)	32 (56%)	
Absent	12 (33%)	14 (65%)	25 (44%)	
Eosinophilic granular bodies				0.01 *
Present	27 (73%)	9 (45%)	36 (63%)	
Absent	10 (37%)	11 (55%)	21 (37%)	
Perivascular lymphocytes				0.70
Present	24 (65%)	13 (65%)	37 (65%)	
Absent	13 (35%)	7 (35%)	20 (35%)	
Microvascular proliferation				
Present	0	0	0	
Absent	37 (100%)	20 (100%)	57 (100%)	
Necrosis				0.0007 *
Present	3 (8%)	8 (40%)	11 (19%)	
Absent	34 (92%)	12 (60%)	46 (81%)	

*p*-Values compare grade 2 and grade 3 tumors. Bold values and * are statistically significant variables.

**Table 3:** Molecular characteristics of the cases of PXA.

Gene/Chromosome Status	PXA G2 n (%)	PXA G3 n (%)	ALL n (%)	*p*-Value
Total number of cases	37	20	57	
*BRAF* V600E mut	30 (81%)	13 (65%)	43 (75.4%)	0.18
*CDKN2A* homo-del	9 (24%)	7 (35%)	16 (28%)	0.4
*IDH1/2* mut	0	0	0	
p*TERT* mut	1 (3%)	7 (35%)	8 (14%)	0.0005 *
Gain +7	8 (22%)	4 (20%)	12 (21%)	0.88
Loss −10	3 (8%)	4 (20%)	7 (12%)	0.19
*EGFR* amp	0	0	0	
*PTEN* del	3 (8%)	5 (25%)	8 (14%)	0.08
*MGMT* methylation	0	2 (10%)	2 (3.5%)	0.051
★Molecular events	4 (11%)	6 (30%)	10 (18%)	0.07

Mut, mutation; amp, amplification; del, deletion. ★ The simultaneous occurrence of ≥three molecular changes in one case is considered as a multimolecular change event in our cohort. *p*-values compare grade 2 and grade 3 tumors. Bold values and * are statistically significant variables.

## Data Availability

Publicly available datasets were analyzed in this study. This data can be found here: https://www.mdpi.com/ethics.
